# From past to progress: a retrospective study on *CFTR* genetic testing in South Africa

**DOI:** 10.1007/s12687-025-00810-6

**Published:** 2025-09-25

**Authors:** Sarah Walters, Colleen Aldous, Helen Malherbe

**Affiliations:** 1 Ampath Laboratories, Centurion, South Africa; 2 School of Clinical Medicine, University of Kwa-Zulu Natal, Durban, South Africa; 3 Centre for Human Metabolomics, Desmond Tutu School of Medicine, Faculty of Health Sciences, University of the North-West, Potchefstroom, South Africa

**Keywords:** Cystic fibrosis, Genetic testing, *CFTR*, Recommendations

## Abstract

Confirming a genetic diagnosis of cystic fibrosis (CF) for clinically affected individuals should be more accessible today, with more laboratories offering testing and improved technologies at lower costs. Instead, diagnostic testing for CF has become more complex due to the variety of genetic testing options available for the one known causative gene (*CFTR*). This article provides an overview of genetic tests currently available for CF in six laboratories in South Africa. Also, it demonstrates the evolution of CF tests used at one private laboratory in the country via a ten-year retrospective study. The findings of this study may serve as a guide for healthcare providers in selecting appropriate testing for CF diagnostic or carrier genetic confirmation. The choice of genetic test and methodology depends on individualised factors such as the ethnic origin of the patient, test availability, advantages and limitations, and cost. The ethnic diversity of South Africa’s populations and probable under-reporting of CF in the country make the diagnosis of this relatively common genetic condition complex. The actual burden of CF in South Africa is unknown, and comprehensive genetic testing, with an ongoing compilation of patient data in the South African CF registry, should assist in addressing the genetic diversity of CF-causing variants.

**Supplementary Information:**

The online version contains supplementary material available at 10.1007/s12687-025-00810-6.

## Introduction

### Cystic fibrosis screening and genetic testing

Cystic fibrosis (CF) is a common, autosomal recessive condition in European-descent individuals, with lower frequencies in other populations (World Health Organization 2004). It can be detected *in utero* or at birth due to symptoms like meconium ileus or failure to thrive, and later in childhood with recurrent respiratory infections and *Pseudomonas aeruginosa* colonisation. Advances in CF-specific modulator therapies have significantly improved life expectancy, increasing from 17 years in 1970 to 53 years in 2021 (Burgener and Cornfield 2023).

While new technology has emerged over the decades globally, recent studies have yet to be published on CF genetic testing currently implemented in South Africa, and little is known about the CF pathogenic variants tested for in South African healthcare.

### South Africa’s healthcare context

**Fig. 1:**
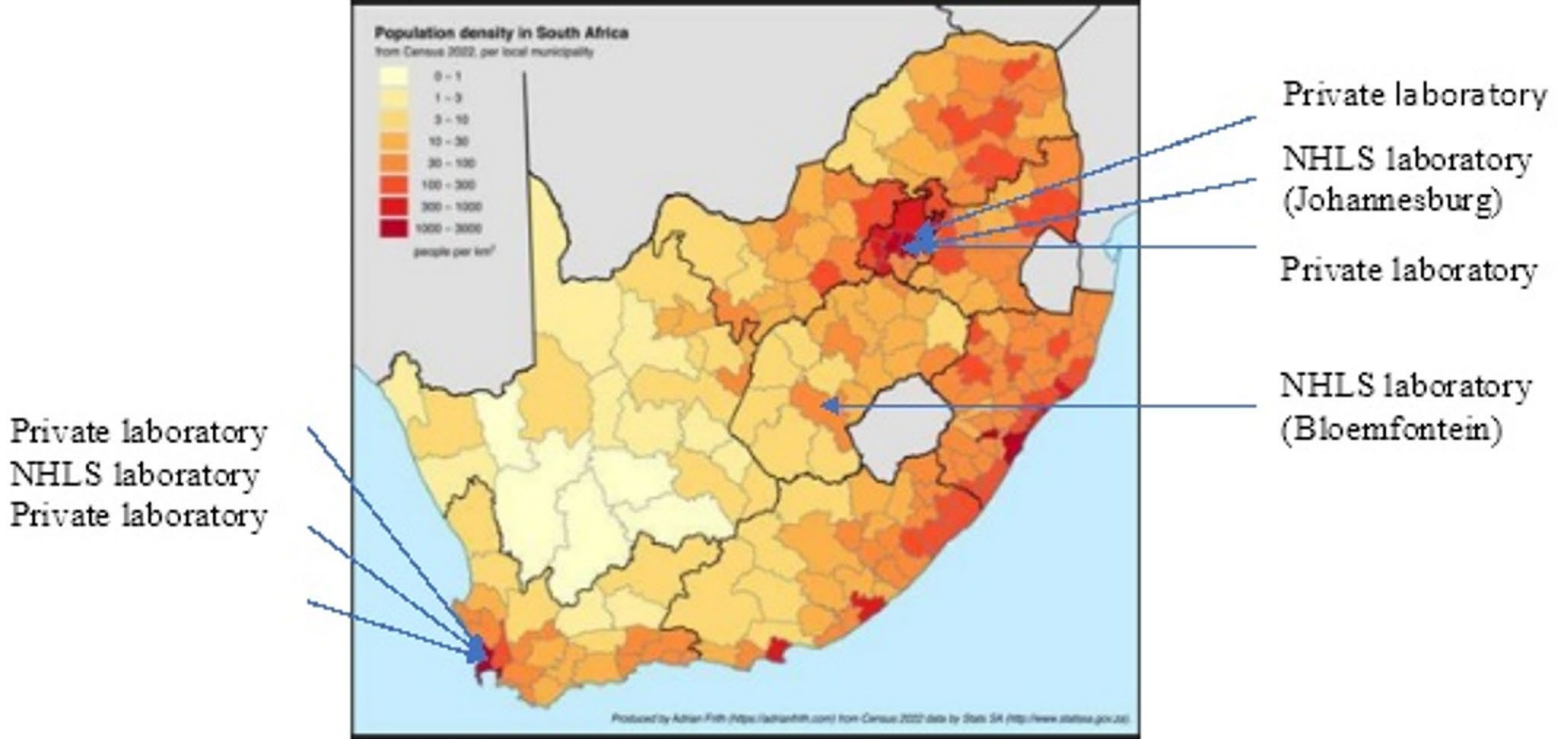
Population density map of South Africa according to the 2022 Census. Locations of diagnostic genetic testing laboratories (private and public NHLS) are indicated. Map produced by Adrian Frith (https://adrianfrith.com) from Census 2022 data produced by Stats SA (http://www.statssa.gov.za). Locations of laboratories included by the authors

South Africa, an upper-middle-income country with a population of 63 million, is ethnically diverse, with 81.7% classified as Black African, 8,5% as Coloured, 7.2% as White, and 2.6% as Indian/Asian (Statistics South Africa 2024). The country currently operates a dual healthcare system. The government-funded public healthcare sector serves approximately 84% of the population (52 million people), while 16% of the population, equating to 9.7 million people, access private healthcare (Cowling 2024). Figure 1 shows the distribution of South Africa’s population and the location of genetic laboratories capable of genetic testing, specifically for CF.

Cystic fibrosis (CF) genetic testing is available in South Africa through the government-subsidised National Health Laboratory Service (NHLS) (National Department of Health 2000), catering to the public sector and private pathology laboratories servicing patients on private healthcare insurance. Public sector testing remains limited, leading to result delays due to batching, reagent availability, lack of capacity, and other challenges.

Sweat chloride testing remains the global standard for CF screening (Farrell et al. 2017; Gibson and Cooke 1959), with genetic confirmation recommended after two positive sweat tests on separate occasions (Mishra et al. 2005; Cowling 2024). Newborn screening (NBS) for CF is available mainly in high-income countries where CF is common but remains largely unavailable in most low- and middle-income countries (LMICs) (da Silva Filho et al. 2021).

The CF transmembrane conductance regulator (*CFTR*) gene responsible for causing CF was identified in 1989 (Kerem et al. 1989), expanding knowledge of CF genetics. The international *CFTR*2 database from Johns Hopkins University, USA (https://CFTR2.org, accessed 13/2/2025) lists 1085 CF-causing variants, 55 variants of varying clinical consequence, and 27 non-CF-causing variants, while ClinVar (https://www.ncbi.nlm.nih.gov/clinvar/, accessed 13/2/2025) reports 5435 *CFTR* variants, including 1165 pathogenic variants plus an additional 500 likely pathogenic variants.

In South Africa, CF testing by linkage analysis was introduced in 1987 (Hitzeroth et al. 1991). It was replaced by targeted testing for the common European population CF pathogenic variant, Delta F508 (also known as ΔF508, or p.Phe508del), later replaced by targeted *CFTR* variant testing (Van Rensburg et al. 2018). Whole *CFTR* sequencing by next-generation sequencing (NGS) and copy number variation (CNV) analysis is now available locally. Modulator therapies were introduced in 2019, dramatically improving clinical outcomes for most patients with CF who are genetically eligible (Graeber and Mall 2023; de Melo et al. 2022).

Treatment for CF has progressed over the decades, focusing on better drugs, nutritional support, and advances in physiotherapy. Genetic confirmation of a CF diagnosis in light of new targeted modulator treatments is essential (de Melo et al. 2022).

CF Globally and in South Africa - carrier frequency and birth prevalence.

While CF carriers occur in all population groups worldwide in varying frequencies, only data for carriers in European and North American populations have been well documented. In individuals of reproductive age, carrier testing is essential to inform reproductive decision-making. Limited data is available for specific countries or population groups in many LMICs. For example, in India, carrier screening is limited to testing for only one common pathogenic variant. This carrier frequency is estimated to be 1 in 238 (Kapoor et al. 2006), while carrier frequencies for CF in Vietnam were reported as 1 in 23 (To-Mai et al. 2024).

The prevalence of CF in all 195 countries worldwide is yet to be documented. Guo et al. (2022) explored the worldwide presence of CF and reported on 94 countries where an estimated 162,428 people live with CF, but a diagnosis was made in only 105,352 of these individuals. These data provide an approximate diagnosed prevalence of between 0.17 and 0.27 affected individuals per 10,000 population. In Africa, nine countries provided an estimated number of 1665 affected individuals, with the majority from Egypt and South Africa, with a prevalence of 0.5 affected individuals per 10,000 population. However, this figure is likely highly under-reported. No information was available in 40 other African countries (primarily LMICs). A review of CF molecular epidemiology in Africa in 2016 showed that 12 of the 49 countries on the African continent have published relevant CF data (Stewart and Pepper 2016).

In South Africa, CF carrier frequencies vary between ethnic groups and were reported in the late 1990s as approximately 1 in 20 for the Caucasian population, 1 in 55 for the Coloured population, and 1 in 34–90 for the Black populations (Padoa et al. 1999). More recent studies indicate the CF birth prevalence as 1 in 3000 in the Caucasian population, 1 in 10,300 in the Coloured population, and 1 in 784 − 13,924 in the Black population (Zampoli et al. 2021b). Both in South Africa and other countries worldwide, a higher carrier frequency of CF and other specific genetic conditions has been documented for the Ashkenazi-Jewish population due to homogeneity and bottlenecks in the population diaspora (Waldman et al. 2022).

A South African Cystic Fibrosis Patient Registry was established in 2018, with the latest report including 505 affected individuals (Zampoli et al. 2024). Based on the reported carrier frequencies in the four South African ethnic groups, CF is still thought to be severely underdiagnosed in the country, especially in the Black population, due to confounding conditions such as malnutrition, TB, and HIV, which may mask CF symptoms (Westwood and Brown 2006). CF may have different disease presentations and phenotypes and may vary between ethnic population groups. Anecdotally, CF is still considered a Caucasian disease in South Africa.

Individuals in the public sector may have limited access to CF testing, including sweat tests and genetic testing, thus delaying treatment due to geographical distance, availability of equipment, healthcare provider awareness, and expertise. Currently, CF genetic testing in the public sector uses targeted variant kits covering pan-ethnic variants in *CFTR* and is therefore limited to reporting specific pathogenic variants only.

Newborn screening for CF in South Africa is currently only available in the private healthcare sector (at parents’ own cost) for families with a CF family history. It is not offered in public healthcare (Zampoli et al. 2021a, Malherbe et al. 2024). As a result, a CF diagnosis generally only occurs after the neonatal period, several weeks or months after birth, thus delaying treatment and allowing disease progression.

Within this South African context, healthcare providers’ awareness of current, appropriate CF genetic testing may need improvement due to new and changing testing methodologies. The aims of this study were: (1) To provide an overview of current and historic genetic tests available/implemented for CF in South Africa across both healthcare sectors; (2) Undertake a ten-year, retrospective study of CF diagnostic testing for private patients undertaken by one of SA’s larger private pathology laboratories, and; (3) Undertake an analysis of healthcare providers requesting these tests.

## Method

The methodology used in this study was an observational descriptive retrospective review of CF genetic test methodologies, genetic results and healthcare provider categories.

1. Current CF genetic tests used in South Africa across public and private healthcare.
  Four private and two of the three public NHLS genetics laboratories were contacted telephonically by the first author in 2019. The lead scientist in each molecular genetics laboratory was asked to specify which CF tests and methodology/kits are used in their laboratory. The two NHLS laboratories included (Johannesburg and Cape Town) represented CF testing in the public healthcare sector (the NHLS genetics laboratory offering CF testing in Bloemfontein was excluded), and these were compared with four private laboratories located in Johannesburg, Cape Town, and Centurion. Once these data were received and anonymised, the pathogenic variants included in each CF testing kit were identified using publicly available information for each manufacturer. These data were collated into a data extraction table adapted from the template in the South African 2017 CF consensus guidelines (Zampoli 2017). See Supplementary File 1 for data points extracted.
2. Retrospective audit of CF tests used in one private genetic laboratory.
  The retrospective review analysed *CFTR* testing undertaken over ten years (January 2013 to December 2022) from one private laboratory in Gauteng. A ten-year timeframe was chosen to illustrate changes and advances in *CFTR* testing during this period and the future potential to improve CF testing for all South African ethnic populations. The private laboratory’s Chief Executive Officer, Chief Operations Officer, and the Head of the Laboratory’s Genetics Department granted gatekeeper permission to access these de-identified data. The head of department internally requested the required data from the laboratory’s data officer via email. These data included the year of testing, test code, age, sex, test results (positive/carrier/negative) and cadre of healthcare provider requesting the test. Descriptive statistics were used to interpret the data. Self-identified population grouping (i.e. ethnicity) is not a data point collected on the laboratory’s test requisition form and, therefore, could not be included.
3. Analysis of healthcare providers requesting CF tests.
  The data collected from the 10-year retrospective study (point 2) above included a code for the cadre of healthcare providers who requested CF testing. This code was converted to the healthcare providers’ qualifications/speciality. The speciality type was documented using Excel^®^ and plotted on a logarithmic scale. The positive, negative, and carrier results were also documented.

Ethical approval for the study was obtained from the UKZN Research Ethics Committee (BFC222/18). De-identified retrospective data were used in compliance with ethical and applicable data protection standards and regulations. No individual could be identified from the anonymised data; therefore, informed consent was deemed unnecessary as per institutional and international ethical frameworks, which permit using anonymised data for research without explicit consent.

## Results

Current and Historic *CFTR* Genetic Testing Methods Used in South Africa:

Data compiled from the six South African laboratories (two public and four private) revealed that four CF kits and whole *CFTR* sequencing were used in the country during the study period. The CF test kits were:

- CF30v2 (Elucigene),
- CFEU2v1 (which replaced CF30v2).
- *CFTR* Core (Devyser AB).
- CF Genotyping Assay (Abbott).

**Table 1:** Current and previous *CFTR* testing methodology implemented at six laboratories in South Africa

Public		Private			
Lab A	Lab B	Lab C	Lab D	Lab E	Lab F
CF30v2 (Elucigene)^1^, CFEU2v1 (Elucigene)^2^	CF30v2 (Elucigene), CFEU2v1 (Elucigene)^2^	2013–2014: ΔF508 2014–2016: CF30v2 (Elucigene)^2^ 2016-current: Whole *CFTR* sequencing + CNV analysis	*CFTR* Core (Devyser)^3^	Previously, *CFTR* sequencing, and now outsourced	CF Genotyping Assay (Abbott)^4^

1. Elucigene^®^ CF30v2 is a gel-based assay used in the French national neonatal CF screening programme. It detects 30 pathogenic variants, most common in the French population

2. Elucigene^®^ CF-EU2v1 is a pan-European CF testing kit to identify 50 pathogenic variants and analyse the poly-T tract. All NHLS laboratories offering kit-based testing utilise the same kit

3. Devyser^®^*CFTR* Core detects the 36 most common pathogenic variants of European origin

4. The Abbott^®^ CF Genotyping assay detects 33 common pathogenic variants as recommended by the American College of Medical Genetics (ACMG) for the “general population”

Fifty-nine pathogenic variants were included in the four kits identified. Information regarding the pathogenic variants in each kit was obtained from the manufacturers (details in Supplementary Table 1).

The CF30v2 kit includes three pathogenic variants common in the Afrikaner population, including 3272–26 A > G, 394delTT, and G542X. The only known common pathogenic variants in the South African Black (c.2988 + 1G > A) and Indian populations (p.Phe508del) were present in all kits. Both p.Phe508del and c.2988 + 1G > A pathogenic variants are also found in the local Coloured population.

### Retrospective study: CF audit of one private laboratory

#### Genetic data and test method evolution

**Fig. 2:**
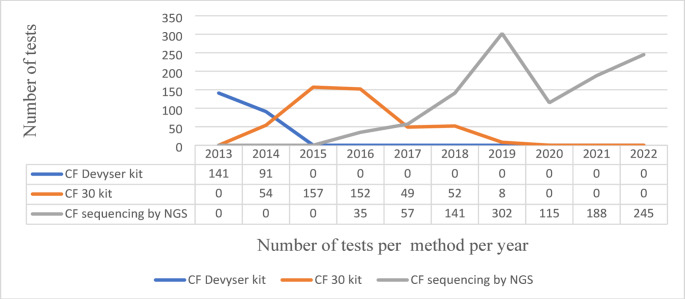
The use of and timing of three *CFTR* testing methodologies in 2013–2022, including the number of samples received for CF testing from Laboratory C (private sector)

The collated data from one private laboratory (Lab C) from 2013 to 2022 showed how CF testing methodology has changed and evolved (Table 1; Fig. 2). Initially, testing was performed using a kit for a limited number of pathogenic variants sent to a referral laboratory (2013–2014). Subsequently, a kit-based method was performed in-house (2014–2019), as outlined in Table 1. Finally, next-generation sequencing (NGS) of the *CFTR* gene (including CNVs) was introduced (2016–2022).

In-house whole *CFTR* sequencing began in 2016 and gradually increased in volume as the benefit of *CFTR* sequencing was realised by referring healthcare providers. The use of targeted variant kits was phased out as sample numbers declined in favour of sequencing.

**Fig. 3:**
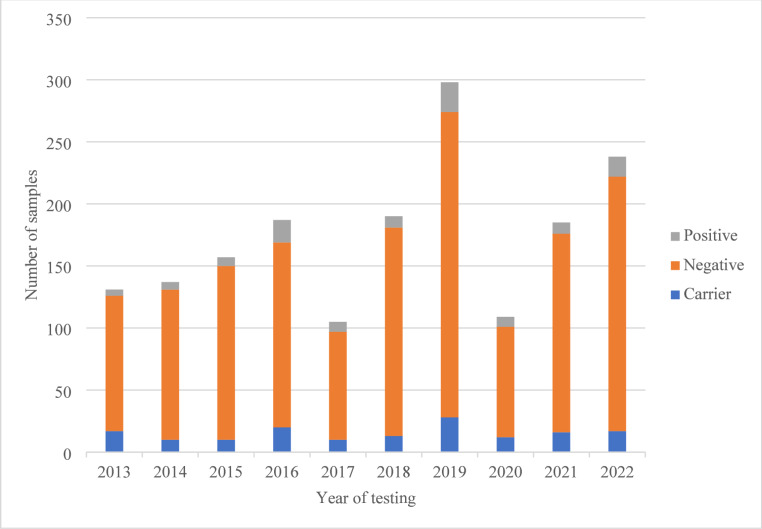
Number of negative, positive, and carrier findings across the retrospective study in Laboratory C

#### Results of patients tested

Over the ten-year study period, 1787 individuals were tested for CF at Laboratory C (Fig. 3). Of these, 13 results could not be traced. Twenty-eight results had one variant of uncertain significance (VUS), and two results had two VUSs. These 43 results (2.4%) were excluded from the analysis, as VUSs cannot confirm or refute a CF diagnosis until future possible reclassification. Of the remaining 1744 available patient results, 108 results (6.2%) had two CF-causing variants and 157 (9%) carrier results (Fig. 2).

The 108 results with two *CFTR* likely/pathogenic variants were compared with the 50 variants detected by the CFEU2v1 test kit used in the public sector. Of these, 16 patients (14.8%) had pathogenic variants which the CFEU2v1 kit would not detect. Of the 157 with carrier results, 97 patients’ carrier results (61.8%) could be identified by the CFEU2v1 test kit. One variant has since been classified as benign, and 15 carrier statuses (15.5%) would not have been detected using the CFEU2v1 test kit methodology.

### Breakdown of referring healthcare providers

**Fig. 4:**
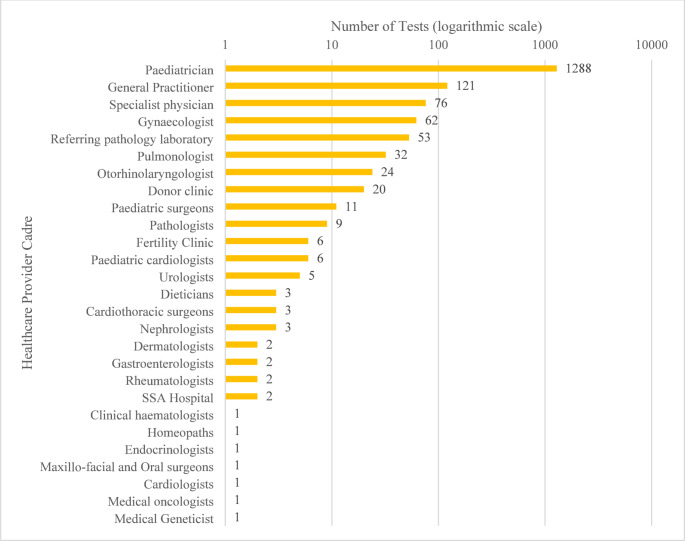
Bar chart showing the number of tests requested by different healthcare provider cadres from the 2013–2022 dataset from one private laboratory in South Africa. Data were converted to a logarithmic scale to include outliers; data labels are actual numbers. See Supplementary File 2

Of the 27 different healthcare provider cadres referring the 1787 individuals for CF testing, the majority were paediatricians (1288/1787, 72%), followed by general practitioners (121/1787, 7%), as shown in Fig. 4 and Supplementary File 2.

## Discussion

This study evaluated available genetic testing for CF patients in South Africa across the public and private sectors, analysing CF testing methodologies used in six laboratories and a decade of CF test results from one private laboratory, including the healthcare practitioners who requested the testing. The detection rate at Lab C over 10 years was 6%, with paediatricians, general practitioners, and specialist physicians being the primary test requestors. This study highlights the role of healthcare providers in making informed decisions when choosing an appropriate CF genetic test for their patients. It emphasises the need for continuous healthcare provider education to stay abreast of new and emerging testing and treatment technologies.

In South Africa, four kit-based methodologies are used, with *CFTR* sequencing introduced in Lab C in 2016. Public-sector CF kit choices are influenced by cost, available technology, and known CF variant profiles in the population, while private labs likely consider similar factors. Given the incomplete understanding of CF genetics across all South African populations, Lab C continues to offer NGS-based *CFTR* sequencing. Complete gene sequencing is essential to improve patient outcomes by identifying all variants and assessing access to the new modulator therapy. This study confirmed 16 CF diagnoses and the identification of 15 carriers, which are not detectable by the kit-based methodology, aiding in treatment decisions and reproductive decision-making.

*CFTR* sequencing is crucial for identifying CF-causing variants in diverse populations and discovering new pathogenic variants. It could inform a tailored, South African-specific kit-based testing approach. Population-based genetic data is essential for improving public health strategies and early CF management in South Africa.

### International recommendations for testing

European recommendations for CF genetic testing at and after birth include primary (population-based targeted testing) and comprehensive (increased number of variants in targeted genetic testing, or *CFTR* sequencing) screening panels (Dodge 2002), both of which are available in South Africa, as shown in Table 1. The American College of Medical Genetics and Genomics (ACMG) recommends that “screening for all CF pathogenic variants at a carrier frequency of more than 1% of a population group is ideal”. This statement also indicates that at least 23 pathogenic variants should be included for CF carrier testing and screening, and that comprehensive methods of *CFTR* testing should be used if necessary (Deignan et al. 2023).

### The South African setting for testing

Ideally, CF genetic testing in South Africa should be based on pathogenic variants found in all the South African ethnic population groups. However, like the global situation, the South African Caucasian population has the most CF genetic data currently available since CF has been diagnosed mainly in this population. CF is thought to be uncommon in the Black, Indian, and Coloured populations, and earlier studies show how a CF diagnosis can be missed in Black patients (Westwood and Brown 2006). CF may also be underdiagnosed in non-Caucasian ethnic groups due to confounding factors, including rare pathogenic variants and varying phenotypes (Westwood ATR 1996). Following international standards for CF testing, most CF pathogenic variants in the Caucasian population are likely to be detected using the kit-based method. *CFTR* sequencing would also be required for affected individuals with only one pathogenic variant, significantly adding to the cost of testing. However, sequencing would be more beneficial as a first-line test in populations with an unknown underlying genetic basis.

### Molecular basis of CF in South Africa

The molecular basis of CF in SA was previously discussed in a 2001 paper (Goldman et al. 2001), including a patient cohort across three of the four ethnic population groups, in which p.Phe508del accounted for 76% of the CF variant of 192 patients, indicating a CF founder effect in the Afrikaner Caucasian population. A further 11 pathogenic variants accounted for 6% of CF chromosomes in the same Afrikaner population. In the same study, approximately 91% of CF-causing pathogenic variants were detected using the CF30v2 kit (based on French population data), also in the Caucasian population (Goldman et al. 2001).

Two pathogenic variants, p.Phe508del and c.2988 + 1G > A; p.? (3120 + 1G > A), were found in this South African Coloured population at frequencies of 43% and 29%, respectively. However, only 14 Coloured and 12 Black patients were included in the study, and the South African Indian population was either excluded or testing had not been requested.

The c.2988 + 1G > A; p.? (3120 + 1G > A) pathogenic variant accounted for 46% of CF pathogenic variants in the South African Black population (Padoa et al. 1999) and was later confirmed by Goldman et al. (2001).

No information could be found on the CF pathogenic variants in the South African Indian population. Prasad et al. (2010) reported that the most frequent CF-causing pathogenic variants in the Indian population in India include p.Phe508del (at 25%, lower than in the European populations), p.Ser549Asn (S549N), c.1525-1G > A, and c.3717 + 12,191 C > T (3849 + 10kbC > T). Further common pathogenic variants in the Indian population are unknown. Collectively, these studies only include a small number of affected individuals. These data add to the support that individuals from these poorly represented populations should have *CFTR* sequencing as a first-line genetic test.

### CF registry data

More recent data from the South African CF Registry indicates that 46% of the 505 individuals included are homozygous for the p.Phe508del pathogenic variant, and 33% are heterozygous (Zampoli et al. 2024). However, this dataset is likely skewed towards excess reports in Caucasian patients, with individuals from other ethnic groups collectively contributing only 32% of the registry dataset (Zampoli, personal communication 2024). These data may not accurately reflect the CF incidence across the different ethnic groups due to the lack of available, accessible genetic testing or the capturing of patient diagnoses from rural areas. Identifying CF carrier frequencies across all population groups is crucial for future screening.

### South African CF testing policy

Aside from recommendations included in the CF consensus guidelines (Zampoli 2017), no equivalent South African governmental nationwide CF testing programme exists compared with those offered in North America, Europe, and other high-income countries. It is recommended that NBS, including screening for CF, is implemented as a starting point to elucidate the CF frequency in the South African populations, as detailed in the 2021 Clinical Guidelines for Genetic Services (Department of Health 2021). The recent World Health Assembly Resolution (WHA) 77.5 of 2024 to accelerate progress towards reducing maternal, newborn, and child mortality in order to achieve Sustainable Development Goal (SDG) targets 3.1 and 3.2 (World Health Assembly 2024) specifies NBS as a crucial implementation action for all member states. NBS is not currently available in the public sector (84% of the population) and may be limited by out-of-pocket costs in the private sector. CF screening needs to be prioritised for implementation countrywide across both healthcare sectors.

### Retrospective study data

The results of the retrospective study at a South African private laboratory (Lab C) show the use of three tests over the ten-year study period. Initially, testing was referred out to another laboratory, and then a kit-based test was introduced in-house, which included the most known pathogenic variants found in European-based populations. During this period, it was realised that more comprehensive testing was required to include all ethnic population groups, which led to the expansion of CF diagnostic testing to *CFTR* sequencing. Sequencing includes screening for all likely/pathogenic variants found across all population groups, including CNVs, and has the potential to identify all CF-causing variants. The CF30v1 kit was introduced in 2014 by Lab C and continued to be used until 2019, overlapping with *CFTR* sequencing until it was validated in 2016. Identifying unique pathogenic variants and copy number variations in the subsequent test results realises the value of *CFTR* sequencing.

Patients tested elsewhere for CF who were known to be clinically affected but in whom only one pathogenic CF variant was detected were retested using *CFTR* sequencing. Before introducing *CFTR* sequencing in South Africa, samples were sent overseas to enable potential participation in CF treatment-related clinical trials ahead of the introduction of CF-modulator therapies. However, since the cost of *CFTR* sequencing is higher than the CF30V2 kit, it is not always the preferred first-line test.

### Healthcare provider cadres requesting CF testing in South Africa

Unsurprisingly, this study found that paediatricians were the healthcare provider cadre requesting the most (74%) CF genetic testing in this cohort, followed by general practitioners (7%). In both the public and private healthcare sectors, paediatricians are at the clinical coalface and are often the first to examine children who may be affected by CF. In South Africa, while genetic counsellors may only request CF genetic testing in collaboration with a clinician, they are a valuable resource for clinicians, patients, and families, advising on appropriate CF genetic testing, taking family histories, and clarifying an individual’s ethnic population through self-identity. While previous studies do not specify the ranking of healthcare provider cadres referring patients for CF tests, they indicate that paediatricians, physicians, gynaecologists and medical geneticists contribute to test referrals, as confirmed by the current study (Baars et al. 2004). However, since paediatricians refer the majority of patients for testing, there is a possibility that other healthcare providers, such as general practitioners and adult pulmonologists, are unaware of the availability of CF testing and would require further education.

### Barriers to CF testing in South Africa

As for many other LMICs, a common challenge in South Africa is the cost of genetic testing equipment, imported reagents, infrastructure, and relevant and adequate expertise/capacity. The public healthcare sector laboratories are currently significantly understaffed and underfunded, leaving many public sector-dependent affected individuals struggling to access a diagnosis and appropriate treatment (Malherbe 2015). Genetic testing is also centralised in three main urban areas: Johannesburg, Cape Town, and Bloemfontein, with limited coverage elsewhere. This creates logistical challenges in terms of distance that either patients or blood samples must travel in order to access CF genetic testing. Since NBS for CF has not yet been included in the public healthcare system, this reduces early identification of the potential number of infants who may be at risk for a CF diagnosis, highlighting a large gap in newborn care.

Continued training of Health Professionals Council of South Africa (HPCSA)-registered medical scientists, renewed political will, and allocation of required resources are necessary. The newly launched Nngwe project (2024) encourages clinicians and scientists to pool rare disease resources (including infrastructure and capacity), data, and knowledge for the greater good of the country’s rare disease community. The country urgently needs increased capacity in sparsely populated and rural areas, including improved, expanded infrastructure and greater expertise among healthcare providers in primary healthcare to identify CF clinical symptoms and improve *CFTR* testing referrals. Additionally, wider availability of CF sweat testing is required countrywide, with adequately trained staff to reduce the possibility of false negative and positive results.

In South Africa, a lack of population-specific demographic data may prevent specific CF pathogenic variants from being identified across all ethnic populations when using kit-based methodology for genetic testing. Once a broader knowledge base regarding specific population groups and CF variants is established, it may be possible to custom-design targeted kits for the country’s specific sub-populations, similar to the CF30 kit developed for the French population. Alternatively, reducing the cost of *CFTR* sequencing may alleviate the population-specific issue of *CFTR* genetic testing, which may be possible through initiatives such as the Nngwe project.

### Impact of CF therapies on CF testing in South Africa

Following the international introduction of CF modulator medications in 2019, healthcare providers in South Africa requested *CFTR* sequencing more frequently to identify pathogenic variants and determine patients’ eligibility to access these new treatments. The 2019 spike in testing in Lab C may be ascribed to CF specialist clinics’ doctors requesting sequencing for patients who either lacked a paper/electronic copy of previous testing reports confirming their CF genetic diagnosis or for those patients with only one known pathogenic *CFTR* variant reported at the time of initial diagnosis when *CFTR* sequencing was unavailable. When *CFTR*-modulator therapies became accessible to identified “genetically eligible” individuals with specific CF variants, a genetic report demonstrating eligibility was also required to accompany the therapy request.

 In South Africa and worldwide, individuals with at least one confirmed p.Phe508del pathogenic variant who are > 2 years old are eligible for triple therapy modulators of elexacaftor-tezacaftor-ivacaftor (Trikafta™) (Vertex Pharmaceuticals Inc 2024). However, most patients cannot access this therapy due to the high cost and the lack of approved alternative reimbursement methods, making access difficult (da Silva Filho et al. 2021). Since April 2024, eligible private CF patients in South Africa with medical insurance can access CF modulator benefits up to R400,000 (approximately $22,000) annually (Spotlight 2024). Only 45% of eligible CF patients in South Africa can access this treatment, excluding most patients (Personal Communication, Dr. M. Zampoli, 28 November 2024). If Trikafta™ becomes more affordable and accessible, there may be an increased demand for CF sequencing in the country to confirm patient eligibility. The most recent FDA-approved modulator therapy, Alyftrek™, also produced by Vertex, has not yet been introduced into the South African market and is not yet available for South African patients.

The need for CF sequencing is also highlighted by Amaral and Harrison (2023), who reported that although *CFTR* modulator therapies are now available, there is a need for further drug development to tackle the basis of CF cellular defects to improve clinical benefits for affected individuals and those with *CFTR*-related disorders. In this way, novel therapies could potentially treat every *CFTR* variant.

Other prospective CF treatments being trialled globally include gene editing by various methods, such as CRISPR-Cas9, or using gene delivery with various vectors in cell models of CF (Lee et al. 2021). Currently, no known human trials are underway. The global challenge is enabling equitable treatment access for all CF patients, regardless of geography, population demographic grouping, or socioeconomic status. The new modulator treatment (Trikafta™) has been a life-changing medication for many affected individuals, but unfortunately, it leaves those with rare CF variants untreated. The International Cystic Fibrosis Foundation’s campaign for CF patients to “leave no patient behind” aligns with the SDG call (World Health Organisation 2024) and encourages academia and the pharmaceutical industry to continue searching for a cure for all CF patients worldwide (Choi and Engelhardt 2021).

### Study limitations

This study included past and current testing methodologies as reported by the six included laboratories only and may not represent all South African laboratories. CF test numbers for the ten-year retrospective audit were restricted to one private pathology group (Lab C), contributing to approximately 40% of the private healthcare market share. While including additional laboratories in the study would offer a more comprehensive overview of testing available today and historically in South Africa, this study serves as a starting point in building the CF testing landscape in the country.

The study’s ten-year review of patient results did not include ethnicity/race-specific data, as this is not a data indicator requested or captured during sample processing. Statistics SA breaks down the South African population into four ethnic categories; this assists in identifying inequity in healthcare services.

This article does not describe the rare combinations of CF pathogenic variants to ensure compliance with the Protection of Personal Information Act (POPIA) (Government of South Africa 2013). These data are anonymised, as rare combinations of variants may lead to the identification of affected person/s from their genetic data due to the small number of individuals affected.

## Conclusion

The burden of disease attributed to CF in South Africa remains unquantified to date, with many babies and children remaining undiagnosed or misdiagnosed due to other confounding illnesses that may interfere with CF early detection and referral. This study has provided the current and historic CF testing approaches used in South Africa for a limited cohort of CF patients. It highlights the role of healthcare providers in making informed decisions when choosing a CF genetic test for their patients, emphasising the need for healthcare providers’ continuous education to stay abreast of new emerging technology (Walters et al. 2024).

For South Africa to fully understand its CF burden, newborn screening is urgently required for births in both the public and the private sectors. The Department of Health needs to implement and enforce newborn screening programmes to elucidate the number of children who will develop CF, so that appropriate early interventions can be made available to those infants and lifelong plans for ongoing treatment. Full *CFTR* sequencing needs to be made available for those individuals and populations where two pathogenic variants are not detected after confirmatory immunoreactive trypsinogen testing is positive. Only then can we have an understanding of the true burden of CF disease in South Africa,

With whole *CFTR* gene sequencing becoming more affordable in South Africa, it is hoped that all children and adults suspected of having CF, regardless of geography, demographic population group, or socioeconomic status, can be tested and referred for treatment. *CFTR* sequencing is a key tool in identifying *de novo* pathogenic variants in South Africa’s ethnically diverse population. Despite having limited capacity, South Africa has the technology to test for all CF-causing pathogenic variants, including carrier testing in both healthcare sectors. A key challenge in the country is to develop more cost-effective CF sequencing testing for widespread roll-out in the public healthcare sector and low-resource settings throughout South Africa.

It is recommended that cascade testing and improved utilisation of genetic counsellors for consultations and interpretation of genetic results should be offered to at-risk family members using targeted testing to identify familial CF carriers. Carrier identification will, in effect, contribute to knowledge of the actual burden of CF in South Africa and ultimately contribute to more South Africans affected by and at risk of CF being referred for genetic counselling and treatment in the country.

## Electronic supplementary material

Below is the link to the electronic supplementary material.

Supplementary Material 1

Supplementary Material 2

## Data Availability

Data provided is not available publicly to protect the identity of the individual according to the South African POPIA Act.
