# Supplementary material for: From past to progress: a retrospective study on CFTR genetic testing in South Africa
Source: J Community Genet. 2025 Sep 25;16(6):827–38. doi: 10.1007/s12687-025-00810-6 (PMC12569260; doi:10.1007/s12687-025-00810-6)
Supplement: Supplementary file 1 — Supplementary Material 1 [file 12687_2025_810_MOESM1_ESM.docx]

Supplementary Table 1:

List of targeted pathogenic variants detected in the three kits used for CFTR testing in SA.

|  |  |  |  |  |
| --- | --- | --- | --- | --- |
| Legacy Name | c.DNA / protein nomenclature | CF30v2 (Elucigene) kit | CFTR Core (Devyser AB) | CF Genotyping Assay (Abbott) |
| Y1092X | 3276C>A / p.Tyr1092X | √ | √ |  |
| 1717-1G>A | c.1585-1G>A/ p.? | √ | √ | √ |
| G542X^1^ | c.1624G>T / p.Gly542X | √ | √ | √ |
| W1282X^1^ | c.3846G>A / p.Trp1282Arg | √ | √ | √ |
| N1303K^1^ | c.3909C>G / p.Asn1303Lys | √ | √ | √ |
| ∆F508^1,3^ | c.1521_1523delCTT / p.Phe508del | √ | √ | √ |
| 3849+10kbC>T^1^ | c.3717+12191C>T/ p.? | √ | √ | √ |
| 394delTT^1^ | c.262_263delTT / p.Leu881IlefsX22 | √ |  | √ |
| 621+1G>T | c.489+1G>T/ p.? | √ | √ | √ |
| S1251N | c.3752G>A / p.Ser1251Asn | √ |  |  |
| G551D | c.1652G>A / p.Gly551Asp | √ | √ | √ |
| R117H | c.350G>A / p.Arg117His |  | √ | √ |
| R1162X | c.3484C>T / p.Arg1162X | √ | √ | √ |
| R334W | c.1000C>T / p.Arg334Trp | √ | √ | √ |
| A455E | c.1364C>A / p.Ala455Glu | √ |  | √ |
| 2183AA>G | c.2051_2052delAAinsG / p.Lys684SerfsX38 | √ | √ | √ |
| 3659delC | c.3528delC / p.Lys1177SerfsX15 | √ | √ | √ |
| 1078delT | c.948delT / p.Phe316LeufsX12 | √ | √ | √ |
| I507del | c.1519_1521delATC / p.Ile507del | √ | √ | √ |
| R347P | c.1040G>C / p.Arg347Pro | √ | √ | √ |
| R553X | c.1657C>T / p.Arg553X | √ | √ | √ |
| E60X | c.178G>T / p.Glu60X | √ |  |  |
| 1811+1.6kbA>G | 1679+1.6kbA>G/ p.? | √ |  |  |
| 3272-26A>G^1^ | c.3140-26A>G/ p.? | √ | √ |  |
| 2789+5G>A | c.2657+5G>A/ p.? | √ | √ | √ |
| 3120+1G>A^2,3^ | c.2988+1G>A/ p.? | √ | √ | √ |
| 711+1G>T | c.579+1G>T/ p.? | √ | √ | √ |
| G85E | c.254G>A / p.Gly85Glu | √ | √ | √ |
| Y122X | c.366T>A / p.Tyr122X | √ |  |  |
| W846X | c.2537G>A / p.Trp846X | √ |  |  |
| CFTRdele2,3(21kb) | c.54-5940_273+10250del21kb / p.Ser18ArgfsX16 |  | √ |  |
| 1898+1G>A | c.1766+1G>A/ p.? |  | √ | √ |
| R560T | c.1679G>C / p.Arg560Thr |  | √ | √ |
| L1077P | c.3230T>C / p.Leu1077Pro |  | √ |  |
| R117C | c.349C>T / p.Arg117Cys |  | √ |  |
| L1065P | c.3194T>C / p.Leu1065Pro |  | √ |  |
| R347H | c.1040G>A / p.Arg347His |  | √ | √ |
| T338I | c.1013C>T / p.Thr338Ile |  | √ |  |
| I336K | c.1007T>A / p.Ile336Lys |  | √ |  |
| 1677delTA | c.1545_1546delTA / p.Tyr515X |  | √ |  |
| 2184insA | c.2052_2053insA / p.Gln685ThrfsX4 |  | √ |  |
| 2143delT | c.2012delT / p.Leu671X |  | √ |  |
| IVS8: 5/7/9T | VARIANT* |  | √ | √ |
| 2184delA | c.2052delA / p.Lys684AsnfsX38 |  |  | √ |
| S549N | c.1646G>A / p.Ser549Asn |  |  | √ |
| S549R | c.1647T>G / p.Ser549Arg |  |  | √ |
| V520F | c.1558G>T / p.Val520Phe |  |  | √ |
| 3876delA | c.3744delA / p.Lys1250ArgfsX9 |  |  | √ |
| 3905insT | c.3773_3774insT / p.Leu1258PhefsX7 |  |  | √ |
| F508C | c.1523T>G / p.Phe508Cys |  |  | √ |
| I506V | VARIANT*: c.1516A>G / p.Ile506Val |  |  | √ |
| I507V | VARIANT*:c.1519A>G / p.Ile507Val |  |  | √ |
| I508C | VARIANT*: c.1523T>G / p.Phe508Cys |  |  | √ |

^1^common in SA White population, including Afrikaner population

^2^ found in SA Black population

^3^common in White and Coloured populations in SA

*Variant is a change in the DNA sequence which has unknown pathogenicity and may contribute towards the phenotype
