# Supplementary material for: From past to progress: a retrospective study on CFTR genetic testing in South Africa
Source: J Community Genet. 2025 Sep 25;16(6):827–38. doi: 10.1007/s12687-025-00810-6 (PMC12569260; doi:10.1007/s12687-025-00810-6)
Supplement: Supplementary file 2 — Supplementary Material 2 [file 12687_2025_810_MOESM2_ESM.docx]

Supplementary table 2

HCP cadres that requested CF genetic testing between 2013-2022 with positive, negative and carrier results. Results with one (n=30) or two VUSs (n=2) or no result available (n=19) are excluded.

| **Row Labels** | **Positive** | **Carrier** | **Negative** | **Total** | **% of the total number of tests requested** |
| --- | --- | --- | --- | --- | --- |
| Paediatrician | 77 | 98 | 1113 | 1288 | 74 |
| General Practitioner | 16 | 23 | 82 | 121 | 7 |
| Specialist physician | 9 | 10 | 57 | 76 | 4.4 |
| Gynaecologist | 1 | 7 | 54 | 62 | 3.6 |
| Referring pathology laboratory | 4 | 5 | 44 | 53 | 3.1 |
| Pulmonologist |  | 2 | 30 | 32 | 1.8 |
| Otorhinolaryngologist | 1 |  | 23 | 24 | 1.4 |
| Fertility Donor clinic |  | 2 | 18 | 20 | 1.2 |
| Paediatric surgeons |  | 1 | 10 | 11 | 0.6 |
| Pathologists |  | 1 | 8 | 9 | 0.5 |
| Fertility Clinic |  |  | 6 | 6 | 0.3 |
| Paediatric cardiologists | 1 |  | 5 | 6 | 0.3 |
| Urologists | 1 | 2 | 2 | 5 | 0.3 |
| Dieticians |  |  | 3 | 3 | 0.2 |
| Cardiothoracic surgeons |  |  | 3 | 3 | 0.2 |
| Nephrologists |  | 1 | 2 | 3 | 0.2 |
| Dermatologists |  |  | 2 | 2 | 0.1 |
| Gastroenterologists |  | 1 | 1 | 2 | 0.1 |
| Rheumatologists |  |  | 2 | 2 | 0.1 |
| SSA Hospital (referral – no HCP cadre reported) |  |  | 2 | 2 | 0.1 |
| Clinical haematologists |  |  | 1 | 1 | 0.05 |
| Homeopaths |  |  | 1 | 1 | 0.05 |
| Endocrinologists |  |  | 1 | 1 | 0.05 |
| Maxillo-facial and Oral surgeons |  |  | 1 | 1 | 0.05 |
| Cardiologists |  |  | 1 | 1 | 0.05 |
| Medical oncologists |  |  | 1 | 1 | 0.05 |
| Medical Geneticist |  |  | 1 | 1 | 0.05 |
| **Grand Total** | **110** | **153** | **1474** | **1737** |  |
